# The annual conference of the Irish College of Ophthalmologists: examining over a decade of trends

**DOI:** 10.1007/s11845-023-03324-7

**Published:** 2023-03-20

**Authors:** Lily Farrell, Khadija Gull, Siobhan Kelly, Colm O’Brien, Louise O’Toole

**Affiliations:** 1 University College Dublin, Dublin, Ireland; 2 The Irish College of Ophthalmologists, Dublin, Ireland

**Keywords:** Gender, Publication rate, Subspecialty, Training

## Abstract

**Background:**

The annual conference of the Irish College of Ophthalmologists (ICO) is a key calendar event for ophthalmology research in Ireland.

**Aims:**

We investigated whether there were identifiable trends across various domains for the last twelve ICO meetings. Our objectives were to assess subspeciality and training centre representation, as well as the characteristics of the first author to include gender and stage of training.

**Methods:**

A retrospective analysis of paper and poster presentations from the ICO annual conference yearbooks was conducted. The representation of subspecialties, affiliated institutions, and gender distribution were noted for both categories. For paper presentations, the author’s career stage, full-text publication rates, and impact factors were also determined.

**Results:**

A total of 306 paper presentations and 306 poster presentations were analysed. The subspecialty of retina had the highest representation within both sections. The overall mean publication rate was 38% (range, 6–39%), with a mean journal impact factor of 2.02. No statistically significant differences in gender noted with regard to poster, paper, or publications (*p* < 0.9, *p* < 0.1, *p* < 0.7, respectively).

**Conclusions:**

This is the first review of all research contributions to the ICO conference. We found that there is a need to promote research in some underrepresented subspecialities and training centres. No significant gender bias was found. There is scope to improve the publication conversion rate; this would allow for greater dissemination of the research presented at the ICO meeting.

## Introduction

The Irish Ophthalmological Society was founded in 1918 and evolved to become the Irish College of Ophthalmologists (ICO) in 1992. The ICO serves as the recognised training organisation for ophthalmologists in Ireland. Ophthalmology is a specialty that has been at the forefront of innovation, with research as a core principle of training schemes across the globe [1]. The annual ICO meeting is a key event for promulgating current research work across all levels of training and all ophthalmic subspecialties in Ireland [2]. It is a conference that attracts international attention, with a growing number of posters and papers presented each year [3]. The most recent ICO meeting held in 2022 saw the highest number of poster and paper presentations in the last 12 years [3].

Due to an ever-growing number of research outputs in the current academic climate across all areas of medicine, it is important to analyse trends in research. Analysis of current research trends is an integral step in tracking progress and impact, identifying existing gaps in research, and informing future directions. Additionally, establishment of such trends allows identification of broader issues such as subspecialties with research gaps, discrepancies in gender distribution, and barriers to publications. Publication rates vary significantly between disciplines [4, 5]. Several studies have shown that a gender disparity, which favours male authorship, exists in ophthalmology [6–8]. To our knowledge, there have been no studies published to date examining either the publication conversion rates, or the distribution of authorship by gender, specifically in the context of Irish ophthalmology.

This study aims to review data from the last twelve annual ICO meetings and to elucidate trends across various domains in the research presented, with a goal to identify underrepresented subspecialties and institutions as well as examining publication rates and any potential gender disparities.

## Methods

### Data retrieval

We conducted a retrospective analysis of data from the last 12 years of the annual ICO conference by reviewing the ICO annual conference yearbooks. The yearbooks were accessed from the ICO website. From these yearbooks, the data were separated into two broad categories consisting of paper and poster presentations. Within both categories, the following data were collected from each abstract; the subspecialty grouping of the presentation, the gender, and the affiliated institution of the first author. For paper presentations, we additionally determined the stage of training of the first author and the subsequent paper publication rate. If a paper was published, the impact factor of the publishing journal was noted. The mean impact factor for all papers published in each calendar year was calculated.

### Gender and career stage

To determine the career stage and the gender of the first author, a list of ophthalmic trainees was requested from the ICO. Various training categories were identified to include basic surgical/medical training (BST, BMT) and higher surgical/medical training (HST, HMT) from 2015 to 2022. The data also included international trainees who were a part of the International Medical Graduate Training Initiative/Programme (IMGTI/IMGTP). Authors who were not enrolled in a structured training programme were listed by their non-consultant hospital doctor (NCHD) stages, namely intern, senior house officer, or registrar. As training schemes varied in structure and selection criteria prior to 2015, data relating to career stage predating this were excluded.

### Subspecialties

Subspecialties were assigned based on the relevance of the study title and abstract to each subspecialty area. The subspecialties were divided into the following categories: anterior segment/external eye disease, uveitis, cataract, retina, glaucoma, ocular oncology, orbit, paediatric ophthalmology, refractive surgery, neuro-ophthalmology, and other. The ‘other’ section included presentations on topics such as quality improvement measures/audits and cost–benefit analyses.

### Publication conversion rate

The conversion rates of paper presentations to full text publications were initially determined using the PubMed databases. As a first step, the abstract titles and author list presented in the ICO yearbooks were used as search terms. Publications that appeared after the search were then matched to those listed in the corresponding yearbook. If this approach failed to yield any results, keywords from the abstracts were used. If no matching results were found in the PubMed database despite these measures, Google and Google scholar search engines were used to search for the research work in question.

A defined criterion was used to determine if a presentation had been successfully published. To classify as a successful publication, the abstract found in the relevant database/search engine must have matched the abstract presented at the ICO meeting in relation to its title, methodology, results, and conclusions. The published abstract must also have included at least one author from the original abstract. Presentations from 2022 were excluded due to the very recent nature of the studies. Once a publication was identified, the impact factor of the journal in which the article was published was recorded. Additionally, the gender of the first author of the publication was noted.

### Data analysis

The raw data were analysed using Microsoft Excel version 16.54 software.

## Results

**Fig. 1:**
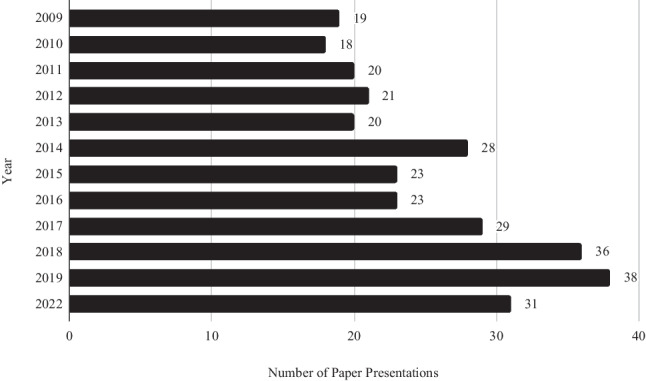
Number of paper presentations per year

**Fig. 2:**
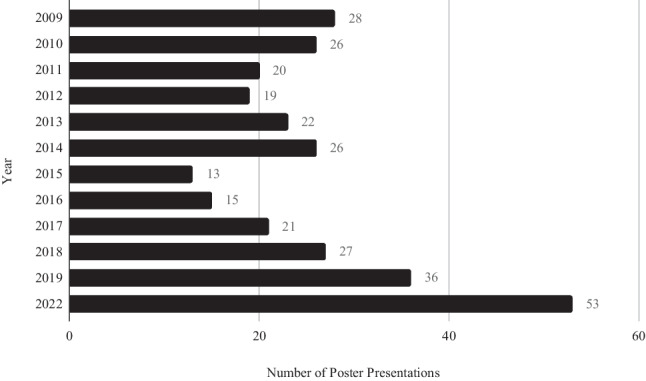
Number of poster presentations per year

A total of 612 abstracts were featured at the ICO Annual Conference between 2009 and 2022. The conference did not proceed in 2020 and 2021 due to COVID-19-related restrictions. Of these 612 abstracts, 306 (50%) were oral paper presentations and 306 (50%) were poster submissions (Figs. 1 and 2).

### Gender distribution

Of the 306 first authors identified in oral presentations, 137 (44.8%) were male and 169 were female (55.2%) (Table 1a). There was no statistically significant difference between the two groups (paired t test, *p* < 0.1). The gender breakdown of the 306 poster submissions identified 145 males (47.4%) and 161 females (52.6%) as first authors (Table 1b). Similarly, there was no statistically significant difference observed between these two groups of authors (*p* < 0.9).

**Table 1:** Gender breakdown of paper presentations

**a**					
**Year**	**Total**	**Males**		**Females**	
		*Number*	*%*	*Number*	*%*
2009	19	9	47	10	53
2010	18	10	56	8	44
2011	20	9	45	11	55
2012	21	5	24	16	76
2013	20	8	40	12	60
2014	28	11	30	17	61
2015	23	7	30	16	70
2016	23	7	59	16	70
2017	29	17	59	12	41
2018	36	17	47	19	53
2019	38	20	53	18	47
2022	31	17	55	14	45

**b**					
**Year**	**Total**	**Males**		**Females**	
		*Number*	*%*	*Number*	*%*
2009	28	16	57	12	43
2010	26	11	42	15	58
2011	20	7	35	13	65
2012	19	9	37	12	63
2013	22	9	41	13	59
2014	26	10	38	16	62
2015	13	8	62	5	38
2016	15	11	73	4	27
2017	21	8	38	13	62
2018	27	9	33	18	67
2019	36	20	56	16	44
2022	53	27	51	26	49

### Career stage

NCHDs undergoing their basic specialist training were consistently at the forefront of paper submissions to the ICO Conference, contributing more than half (55%) of presentations (Table 2). The consultant body was less likely to present papers, with a mean rate determined of 6% (range, 0–13%). The career grade was not known for several authors (range, 0–31%).

**Table 2:** Stage of training of paper first authors (percentage per annum)

**Year**	**Total**	**NS**	**BMT**	**HMT**	**BST**	**HST**	**IMGTI**	**Consultant**	**Orthoptics**	**Unknown**
2016	23	26	0	0	43	18	4	0	0	0
2017	29	7	0	0	52	7	0	0	3	31
2018	36	6	3	0	60	28	3	0	0	0
2019	38	11	5	3	19	26	5	8	5	18
2022	31	16	7	7	15	19	0	13	0	23

*NS* non-scheme, *BMT* basic medical training, *HMT* higher medical training, *BST* basic surgical training, *HST* higher surgical training, *IMGTI* International Medical Graduate Training Initiative/Programme

### Subspecialty breakdown

The three leading areas of research represented amongst paper submissions at the ICO Conference stemmed from the retinal (24.2%), glaucoma (15.4%), and anterior segment (14.7%) subspecialties, respectively (Table 3a). In addition, on year-by-year analysis of paper presentations, retinal-related research contributions have proven to be the most prolific*.*

**Table 3:** Percentage of paper presentations by subspecialty

**a**														
**Year**		**2009**		**2010**	**2011**	**2012**	**2013**	**2014**	**2015**	**2016**	**2017**	**2018**	**2019**	**2022**
**Subspecialty (%)**														
Anterior segment		16		0	25	10	19	11	13	26	15	19	16	7
Glaucoma		11		17	35	0	14	17	17	13	10	14	13	22
Retina		26		0	10	14	19	25	26	35	45	25	32	15
Cataract		16		22	5	19	9	18	10	4	7	8	0	10
Orbit		0		0	0	14	4	0	4	0	3	6	5	0
Neuro*		5		0	5	14	0	4	0	0	7	11	5	0
Refractive surgery		5		6	5	0	15	7	13	0	0	0	0	3
Paediatric		16		11	5	0	15	11	0	8	10	8	5	7
Ocular oncology		0		0	5	5	5	4	4	5	0	6	8	7
Other		5		44	5	24	0	3	13	9	3	3	16	29
**Absolute total**		19		18	20	21	20	28	23	23	29	36	38	31

**b**														
**Year**		**2009**		**2010**	**2011**	**2012**	**2013**	**2014**	**2015**	**2016**	**2017**	**2018**	**2019**	**2022**
**Subspecialty (%)**														
Anterior segment		16		0	25	10	20	11	13	26	14	20	16	7
Glaucoma		11		17	35	0	15	18	17	13	10	14	13	23
Retina		27		0	10	14	20	35	39	40	56	21	30	23
Cataract		14		12	5	0	14	0	24	0	0	6	3	8
Orbit		0		4	5	0	14	4	8	7	5	0	6	2
Neuro*			4	12	10	11	5	15	15	6	0	18	8	9
Refractive surgery		7		0	0	5	0	0	0	0	5	0	0	0
Paediatric		7		4	0	11	9	15	0	7	10	0	6	6
Ocular oncology		0		8	0	0	9	0	8	0	0	4	8	0
Other		32		19	15	36	0	12	0	20	24	33	28	37
**Absolute total**		28		26	20	19	22	26	13	15	21	27	36	53

^*^ *Neuro*, neuro-ophthalmology

On analysis of the poster results, retina had the greatest representation across all years analysed. Posters featuring anterior segment research were less prominent since 2013 (Table 3b). Overall, there was greater discrepancy between contributions from various subspecialties in the category of posters when compared to paper presentations.

### Affiliated institutions

Analysis of the ICO Conference yearbooks revealed contributions from over 35 different institutions both nationally and internationally over the time frame examined. The 10 most consistent contributors to the ICO Conference were identified, The Royal Victoria Eye and Ear Hospital (RVEEH) (30.7%) and the Mater Misericordiae University Hospital (MMUH) (23.5%) contributed most strongly to the oral paper presentations at the conference during the time frame examined (Table 4a). Similar to the paper results, the RVEEH (15.4%) and MMUH (15.0%) contributed the highest number of poster contributions to the conference. Sligo, Waterford, and Cork University Hospitals greatly increased their contribution to the ICO conference since 2015 (Table 4b).

**Table 4:** Affiliated institutions by percentage for paper presentations

**a**												
**Year**	**2009**	**2010**	**2011**	**2012**	**2013**	**2014**	**2015**	**2016**	**2017**	**2018**	**2019**	**2022**
**Total** **%**	19	18	20	21	29	29	39	48	21	28	26	32
**RVEEH**	21	33	20	29	50	28	39	48	21	27	26	31
**MMUH**	16	0	15	29	35	25	26	26	24	17	26	36
**UHW**	11	0	5	0	0	0	0	9	7	11	8	3
**SUH**	0	0	0	0	5	0	0	0	10	3	8	3
**CUH**	0	0	0	5	0	0	4	0	10	3	3	3
**UHL**	5	0	10	0	0	0	4	4	0	6	3	7
**UHG**	0	0	0	0	0	11	13	9	14	11	8	7
**BH**	4	0	5	5	0	4	0	0	0	3	0	0
**TSH**	5	0	0	0	5	4	0	0	0	3	3	0
**OLCHC**	0	0	0	0	5	4	0	0	4	6	0	3
**Other**	38	67	45	32	0	24	14	4	10	10	15	7

**b**												
**Year**	**2009**	**2010**	**2011**	**2012**	**2013**	**2014**	**2015**	**2016**	**2017**	**2018**	**2019**	**2022**
**Total** **%**	28	26	20	19	22	26	13	15	21	27	36	53
**RVEEH**	21	12	10	37	32	15	0	19	0	11	6	19
**MMUH**	4	23	5	11	9	23	15	0	10	22	31	13
**UHW**	4	0	5	0	0	0	31	6	5	19	3	6
**SUH**	0	0	5	0	0	0	8	7	5	4	6	8
**CUH**	11	8	5	5	0	4	0	7	24	19	17	19
**UHL**	0	4	0	0	5	0	0	12	0	0	3	4
**UHG**	11	8	20	0	0	12	15	12	0	11	17	11
**BH**	7	0	0	0	0	0	8	0	10	0	3	0
**TSH**	4	4	0	0	9	8	0	7	0	0	0	2
**OLCHC**	4	4	0	11	5	4	0	0	5	0	0	0
**Other**	34	37	50	36	40	34	23	30	41	14	14	18

*RVEEH* Royal Victoria Eye and Ear Hospital, *MMUH* Mater Misericordiae University Hospital, *UHW* University Hospital Waterford, *SUH* Sligo University Hospital, *CUH* Cork University Hospital, *UHL* University Hospital Limerick, *UHG* University Hospital Galway, *BH* Beaumont Hospital, *TSH* Children’s Health Ireland at Temple Street, *OLCHC* Children’s Health Ireland at Crumlin

### Paper to publication conversion rates

**Fig. 3:**
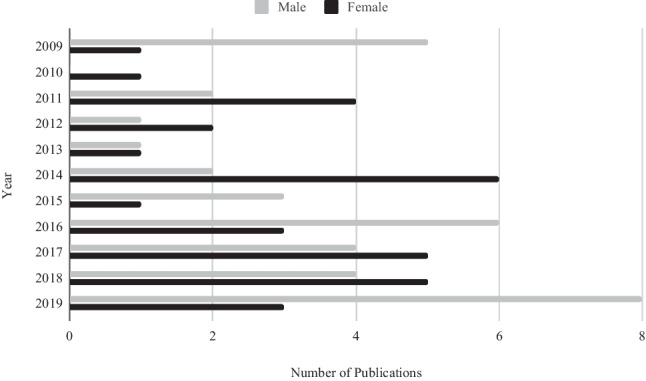
The gender breakdown for published paper presentations by year

The mean rate of paper publication from the ICO Conference for the time frame examined was 24% (range, 6–39%) (Table 5). Since 2016, the percentage of publications reached grew as did the respective average impact factors of their journals (Table 6). There was no statistically significant difference between the gender of published first authors (*p* < 0.7) (Fig. 3).

**Table 5:** Publication rates of paper presentations

**Year**	**Total**	**Papers published %**
2009	19	32
2010	18	6
2011	20	30
2012	21	14
2013	20	7
2014	28	29
2015	23	17
2016	23	39
2017	29	38
2018	36	25
2019	38	32

**Table 6:** Annual impact factor for published paper presentations

**Year**	**Mean impact factor**
2009	1.66
2010	3.52
2011	1.15
2012	0.6
2013	0.3
2014	2.6
2015	3
2016	2.35
2017	1.73
2018	2.31
2019	2.97

## Discussion

This paper serves as the first review of all contributions to the ICO Annual Conference. Results gained from this retrospective study give a clear overview of the research trends in Irish ophthalmology over the last 12 years. The number of paper presentations at the conference grew annually, and the 2022 ICO Conference saw a record number of 53 poster presentations. This study has identified underrepresentation of some subspecialties and training units at the ICO meeting. There was no significant difference seen in the number of presentations at the ICO when accounting for gender. This was true for both poster and paper presentations presented. No gender bias was also observed with respect to the subsequent paper publication rate.

Ophthalmology has a wide and diverse range of subspecialties. The ICO Conference does not have an annual quota that limits nor demands a given number of annual submissions from each subspecialty. Therefore, conference presentations offer a non-biased summary of ongoing clinical research in Ireland. Every abstract is assessed and ranked by each of the members of the ICO Scientific Committee as to its suitability and standard. The number of presentations is generally only capped for papers. A transfer from paper to poster presentation can be facilitated for unsuccessful paper presentations once the abstract is of an appropriate standard. It is important to acknowledge the subspecialties that have been at the forefront of research in Ireland for over a decade as well as to highlight subspecialties where research output may be improved. Our study showed retinal-related research to be an area of major interest at the ICO Conference over the time frame examined with a consistently high level of annual contributions. This mirrors the findings of many other national ophthalmology conferences globally [9–11], suggesting that there is a growing international interest on the management of retinal diseases [12–14]. Glaucoma-related research is also a prominent subspecialty featured at the conference, with a focus on the molecular makeup underpinning the disease process [15, 16]. The anterior segment as a subspeciality has been well represented in paper presentations but less so in the poster section. Neuro-ophthalmology has consistently been underrepresented amongst oral paper presentations at the ICO Annual Conference.

Gender equity in the workplace is an important issue of contemporary discussion. A recent UK study comparing surgical specialties in relation to the gender distribution of trainees showed that ophthalmology had the highest percentage of female trainees [17]. A similar study conducted in Japan highlighted ophthalmology as the third highest specialty in terms of female representation [18]. It is extremely encouraging that females in ophthalmology are so highly represented as such cannot be supported by many other surgical subspecialties [19]. Unfortunately, on a worldwide scale, females still currently only account for approximately 35–45% of ophthalmology trainees [20], with even less representation seen across the USA [21]. However, focusing on our Irish perspective, it is evident that females are well represented at the ICO Conference, with no demonstrable gender bias.

The ICO Conference’s average paper publication conversion rate of 24.4%, although following a growing trend in recent years, falls below the publication conversion rate of several comparable National Ophthalmology Conferences. Okonkow et al. analysed publication rates from the RCOphth Annual congress and revealed a UK ophthalmic publication rate of 44.5% over an 8-year time frame [22]. A similar analysis of the Scottish Ophthalmic Club National Meetings reported a conversion rate of 37% over 5 years [23]. Additionally, a global-average paper-to-publication rate of 38% calculated across 11 International Ophthalmology Conferences has recently been added to the literature [10]. This is definitely an area in need of review so that ICO Conference paper presentations wield a greater impact on the wider ophthalmic community. However, it is promising to see that the ICO Conference mean annual journal impact factor is following a positive trend over the last 6 years. Wenke et al. demonstrated that a short-term, supported funding initiative resulted in more favourable outcomes in relation to individual research capacity and research output from allied healthcare professionals [24]. They found a statistically significant improvement in the securing of funding, qualitative data analysis, and academic writing (*p* < 0.05) after the implementation of this initiative. Optimising clinician-led research is the key to the advancement of treatment options and outcomes in ophthalmology as they are in a unique position of identifying the most pertinent research questions that can benefit patients [25].

Prior to 2018, The National Ophthalmology Training Scheme consisted of a single surgical pathway. This is heavily reflected by the paper results at the ICO Conference where basic surgical trainees (BST) led the way. In 2018, a second strand was introduced—The Medical Pathway, with trainees classified as BMT/HMT. It is encouraging to see several ICO Conference contributions from medical trainees since 2018, which will undoubtedly rise in years to come as this training scheme strengthens and expands. Growing ICO conference additions from non-scheme members are also extremely noteworthy; they highlight the high calibre of candidates awaiting acceptance onto the ophthalmology schemes. Presentation at the ICO meeting allows them to demonstrate their dedication and enthusiasm to the ophthalmic field. The Dublin training hospitals receive the highest quota of trainees, which accounts for the higher number of posters and presentations from these institutions.

We recognise this study had limitations. Data in relation to the stage of training were unavailable for review prior to 2015, which meant that not all authors could be classified based on their respective training stages. Additionally, the poster to publication conversion rate was not analysed in this review. In light of this, our cited publication conversion rate figure, which was derived solely from the paper presentations, is likely to be inflated compared to other studies, as poster presentations tend to have lower presentation-to-publication conversion rates. Finally, secondary to COVID-19, the ICO conference was cancelled in 2020 and 2021, disrupting and limiting research, which may have served to alter 2022 contributions and, thus, results.

Based on the outcomes of our study, we have devised some recommendations. We would suggest surveying those who previously participated in the ICO meeting but did not proceed to publish, as to what barriers they perceived to be present and how they could be overcome. To encourage more research output and publications in the years to come, we would recommend that dedicated research mentoring be available for junior trainees. The concept of nationally protected research days would be useful to facilitate trainees in balancing clinical practice and research projects at work. For eye units to continue to provide a comprehensive ophthalmology service, it is important for all subspecialties to remain attractive to trainees. The ICO meeting offers such a platform for clinician-led research to be presented across all subspecialities of ophthalmology.

## Conclusion

This paper serves as the first objective review of all research contributions to the ICO Annual Conference over the past 12 meetings. The subspecialties with the greatest contributions to the conference were retina, glaucoma, and anterior segment. In relation to research output, BST was the most prolific group. No obvious gender disparities were identified from the data that were analysed with respect to presenting or publishing authors. The ICO Conference average publication conversion rate of 24.4% has shown to be an area with scope for improvement. Aiming for a higher publication conversion rate will allow matching of our research output level to the contributions of other national conferences to the literature in the field of ophthalmology.
